# Comparative rates of upstaging and upgrading in Caucasian and Korean prostate cancer patients eligible for active surveillance

**DOI:** 10.1371/journal.pone.0186026

**Published:** 2017-11-14

**Authors:** Hwang Gyun Jeon, Jae Ho Yoo, Byong Chang Jeong, Seong Il Seo, Seong Soo Jeon, Han-Yong Choi, Hyun Moo Lee, Michelle Ferrari, James D. Brooks, Benjamin I. Chung

**Affiliations:** 1 Department of Urology, Samsung Medical Center, Sungkyunkwan University School of Medicine, Seoul, Korea; 2 Department of Urology, Stanford University Medical Center, Stanford, CA, United States of America; National Health Research Institutes, TAIWAN

## Abstract

**Purpose:**

To investigate the impact of race on the risk of pathological upgrading and upstaging at radical prostatectomy (RP) in an Asian (Korean) and Western (Caucasian) cohort eligible for active surveillance (AS).

**Materials and methods:**

We performed a retrospective cohort study of 854 patients eligible for AS who underwent RP in United States (n = 261) and Korea (n = 593) between 2006 and 2015. After adjusting for age, PSA level, and prostate volume, we utilized multivariate logistic regression analysis to assess the effect of race on upgrading or upstaging.

**Results:**

There were significant differences between Caucasian and Korean patients in terms of age at surgery (60.2 yr. vs. 64.1 yr.), PSA density (0.115 ng/mL/mL vs. 0.165 ng/mL/mL) and mean number of positive cores (3.5 vs. 2.4), but not in preoperative PSA values (5.11 ng/mL vs. 5.05 ng/mL). The rate of upstaging from cT1 or cT2 to pT3 or higher was not significantly different between the two cohorts (8.8% vs. 11.0%, P = 0.341). However, there were higher rates of upgrading to high-grade cancer (Gleason 4+3 or higher) in Korean patients (9.1%) when compared to Caucasian counterparts (2.7%) (P = 0.003). Multivariate logistic regression analysis showed that age (OR 1.07, P < 0.001) and smaller prostate volume (OR 0.97, P < 0.001), but not race, were significantly associated with upstaging or upgrading.

**Conclusions:**

There were no differences in rates of upgrading or upstaging between Caucasian and Korean men eligible for active surveillance.

## Introduction

Over the past 5 years, active surveillance for low risk prostate cancer has gained widespread acceptance in the United States and European countries [1, 2]. The recommendation for active surveillance is predicated on an understanding of the underlying natural history of low risk prostate cancer in the population and is now based on the outcomes of several observational studies [3–5] as well as outcomes from prospective screening trials such as the Prostate, Lung, Colon, and Ovary (PLCO) [6] and European Randomized Study of Screening for Prostate Cancer (ERSPC) [7]. However, the natural history of prostate cancer in Asian men in general, and Korean men specifically, is less well defined. For example, population-based studies have shown conflicting results regarding the relative outcomes between Caucasian and Asian patients. Surgical series have shown higher rates of upgrading in Asian men with low risk prostate cancer compared to Caucasians, although differences in practice patterns, surgical techniques, pathological evaluation, screening practices, and other factors make it difficult to directly compare studies [8, 9]. Furthermore, an emerging body of evidence suggests that there are differences in germline risk alleles as well as somatic genetic changes in tumors in Asians compared to Caucasians.

Given the uncertainty surrounding prostate cancer risk in an Asian population, AS has been less widely accepted in Asian countries compared to the West. However, it is possible that more adverse pathological findings in Asian men compared to Caucasians are due to other factors including higher age and smaller prostate sizes in the populations studied [10–12]. To date, no study has directly compared pathologic upgrading or upstaging in Asian and Caucasian men undergoing radical prostatectomy after adjusting for PSA, prostate volume, and age. To address whether Asian race affects adverse pathological upgrading or upstaging in low risk patients, we compared surgical pathology outcomes in men who qualified for active surveillance in two large radical prostatectomy series.

## Materials and methods

### Patients

This retrospective study was approved by the Institutional Review Board in Samsung Medical Center 2016-03-111. The need for informed consent from patients was waived by the Institutional Review Board because this was a retrospective study. Between January 2006 and August 2015, a total of 4,101 patients underwent radical prostatectomy for prostate cancer at two centers (Samsung Medical Center (n = 2813) and Stanford Medical Center (n = 1,288). Patients who had received hormonal therapy prior to radical prostatectomy and, at Stanford Medical Center, those who were of African American, Hispanic, or Asian ethnicity were excluded. As the Gleason scoring system was updated by the International Society of Urological Pathology in 2006, this was set as the start date for inclusion.

Candidates were designated as being eligible for AS criteria (University of Toronto [13], clinical stage T1c/T2a, PSA level 10 ng/ml or less, and Gleason score 6 or less) on 12-core biopsy. Of the 4101 patients who underwent radical prostatectomy, there were 854 patients who met eligibility criteria for AS who underwent radical prostatectomy (Stanford Medical Center, n = 261, and Samsung Medical Center, n = 593).

A combined dataset of clinical and pathological data was generated for analysis and included age at surgery, pre-operative serum PSA level, prostate volume, PSA density, number of positive cores, biopsy Gleason score, pathologic stage, and pathologic Gleason score. All prostate specimens were reviewed by experienced genitourinary pathologists at each center and pathologic staging was based on the 2010 American Joint Committee on Cancer TNM staging system. After radical prostatectomy, upstaging was defined as pT3 or higher and upgrading was defined as Gleason 4+3 or higher.

### Statistical analyses

Statistical analysis was performed by the chi-square test and independent t test for categorical and continuous variables, respectively. After adjusting for confounders (age, PSA level, prostate volume, and number of positive biopsy cores), we used multivariable logistic regression analysis to assess racial differences in upgrading and upstaging. A separate analysis was performed for 419 patients (Stanford Medical Center, n = 103 and Samsung Medical Center, n = 316) eligible for more strict AS criteria according to PRIAS criteria (clinical stage T1c/T2a, PSA level 10 ng/ml or less, and Gleason score 6 or less, PSA density < 0.2 ng/mL/cm^3^, and no more than two positive cores) [14]. A *p* value < 0.05 was considered statistically significant. All statistical analyses were performed with IBM SPSS version 20.0 (IBM Company, NY).

## Results

From the entire radical prostatectomy cohort of 4101 patients, 261 Caucasian and 593 Korean patients were included in the analysis who met the criteria for AS. There were several notable differences between the Korean and Caucasian men as summarized in Table 1. Korean men were diagnosed with prostate cancer at an older mean age of 64.1 years compared with Caucasian men at 60.2. (P < 0.001). Prostate volume was lower in Korean men than in Caucasian men (35.6 ml vs 51.3 ml, P < 0.001) and PSAD was higher (0.165 vs 0.115, respectively, P < 0.001). The mean number of positive cores was higher in Caucasian men compared to Korean men (3.52 vs 2.44, P < 0.001). The preoperative serum PSA level was not significantly different between the two groups.

**Table 1:** Patient characteristics.

	Caucasian (n = 261)	Asian (n = 593)	P value
Age, yrs., mean (SD)	60.2 (6.4)	64.1 (7.1)	<0.001
PSA level, ng/ml, mean (SD)	5.11 (2.00)	5.05 (1.93)	0.685
Prostate volume, ml, mean (SD)	51.3 (20.3)	35.6 (16.0)	<0.001
PSAD, ng/ml2, mean (SD)	0.115 (0.11)	0.165 (0.16)	<0.001
Biopsy cores sampled, mean (SD)	13.1 (2.1)	12.1 (0.5)	<0.001
Positive cores, mean (SD)	3.52 (2.5)	2.44 (1.9)	<0.001
Pathologic stage			0.341
T2	238 (91.2%)	528 (89.0%)	
T3 or higher	23 (8.8%)	65 (11.0%)	
Prostatectomy Gleason score			<0.001
6	140 (53.6%)	230 (38.8%)	
7 (3+4)	114 (43.7%)	309 (52.1%)	
7 (4+3)	7 (2.7%)	34 (5.7%)	
8 or more	0	16 (4.0%)	
Positive margin, n (%)	47 (18.2)	52 (8.8)	<0.001

SD = standard deviation

Pathological outcomes after radical prostatectomy are summarized in Table 1. Rates of upstaging to ≥ T3 were not significantly different between Caucasian and Korean patients (8.8% vs 11.0%, P = 0.341). However, there were higher rates of significant upgrading (Gleason score ≥ 4+3) in Korean patients (9.1%) when compared to their Caucasian counterparts (2.7%) (P = 0.003). Positive surgical margin rates were lower in Korean (8.8%) prostatectomy specimens compared to those from Caucasians (18.2%) (P < 0.001).

To understand the clinical and pathological features associated with upgrading and upstaging, we performed multivariable logistic regression analysis (Table 2). Older age (OR = 1.07, P < 0.001), higher pre-operative PSA (OR = 1.26, P < 0.001), smaller prostate volume (OR = 0.95, P < 0.001), and a greater number of positive cores (OR = 1.14, P = 0.008) were significant predictors for upstaging (≥ pT3). Older age (OR = 1.07, P < 0.001) and smaller prostate volume (OR = 0.95, P < 0.001) were associated with significant upgrading (Gleason score ≥ 4+3). However, in the multivariable model, Korean men did not have a significantly higher risk of advanced-stage prostate cancer (OR = 0.60, P = 0.139) or pathologic high-grade prostate cancer (OR = 1.18, P = 0.727) compared to Caucasian men.

**Table 2:** Multivariable logistic regression analysis for advanced stage or high-grade cancer.

	Advanced stage (pT3 or higher)		High-grade cancer (Gleason score ≥ 4+3)	
	OR (95% CI)	P value	HR (95% CI)	P value
Age (continuous)	1.07 (1.03–1.11)	<0.001	1.07 (1.03–1.11)	<0.001
PSA (continuous)	1.26 (1.12–1.41)	<0.001	1.13 (0.99–1.29)	0.063
Prostate volume (continuous)	0.95 (0.93–0.97)	<0.001	0.95 (0.93–0.97)	<0.001
Positive core number (continuous)	1.14 (1.06–1.25)	0.008	0.90 (0.77–1.05)	0.202
Race, Korean vs. Caucasian	0.60 (0.31–1.17)	0.139	1.18 (0.46–2.98)	0.727

To evaluate whether Korean vs. Caucasian ethnicity influenced upgrading or upstaging in a very low risk population we performed multivariable logistic regression analysis using a subset of patients selected using PRIAS AS criteria (Table 3). In this analysis of 419 patients (Stanford Medical Center, n = 103 and Samsung Medical Center, n = 316), only smaller prostate volume (OR = 0.96, P = 0.040) was a significant predictor of upstaging (≥ pT3). Older age (OR = 1.08, P = 0.017), higher preoperative PSA level (OR = 1.51, P = 0.003), and smaller prostate volume (OR = 0.91, P < 0.001) were significant predictors for high-grade cancer (Gleason score ≥ 4+3). Once again, Korean men did not have a higher risk of upstaging (OR = 0.46, P = 0.170) or upgrading (OR = 1.01, P = 0.970) compared to Caucasian men.

**Table 3:** Multivariable logistic regression analysis for advanced stage or high-grade cancer in patients with very low-risk prostate cancer.

	Advanced stage (pT3 or higher)		High-grade cancer (Gleason score ≥ 4+3)	
	OR (95% CI)	P value	HR (95% CI)	P value
Age (continuous)	1.05 (0.98–1.12)	0.131	1.08 (1.01–1.16)	0.017
PSA (continuous)	1.14 (0.88–1.48)	0.301	1.51 (1.14–1.98)	0.003
Prostate volume (continuous)	0.96 (0.92–0.99)	0.040	0.91 (0.87–0.96)	<0.001
Positive core number, two vs. one	1.87 (0.80–4.34)	0.144	0.66 (0.25–1.56)	0.666
Race, Korean vs. Caucasian	0.46 (0.15–1.38)	0.170	1.01 (0.25–4.00)	0.979

## Discussion

In a direct comparison of a large Korean cohort with a Caucasian cohort of men undergoing radical prostatectomy, we found that Asian race was not associated with increased risk in upgrading or upstaging in men who met the criteria for active surveillance. When we controlled for age at diagnosis, serum PSA levels, prostate size, and number of positive cores, race was not associated with the risk of upgrading and upstaging. This finding suggests that in properly selected patients, AS is a potentially safe option for management of prostate cancer, regardless of Asian ethnicity.

Clinical, pathological, and biological characteristics of prostate cancer have been reported to vary according to ethnic background. It has long been known that African American men have higher rates of prostate cancer incidence and death compared to Caucasians [15, 16]. With regard to Asians and Asian-Americans, the data is seemingly conflicting with lower overall incidence rates [17] and reportedly worse clinical and pathologic features at the time of diagnosis when compared to Caucasians [18, 19]. A recent study demonstrated that compared to Caucasians, Asian-American men were more likely to show unfavorable risk profiles at the time of diagnosis, comparable to those observed in African Americans [20]. In agreement with findings of more aggressive disease in Asian men, rates of pathological upgrading or upstaging in radical prostatectomy series in Asian men who would meet criteria for active surveillance are higher compared to those reported in Caucasian men, ranging from 44–54% in Korean men [8] and 27–51% in Japanese men [9], compared to the approximately 20–34% rate in Western men [10, 21]. Recently, Jeong et al reported the percentage of upstaging and upgrading in 700 patients with low-risk prostate cancer (PSA <10 ng/ml, cT1 stage, and biopsy Gleason score 6) in both Koreans and Caucasians. Both the percentage of upstaging to advanced-stage prostate cancer (pT3 or higher) and upgrading to high-grade prostate cancer were higher in Korean men than in Caucasian men, even after adjusting for age, PSA level, prostate volume, and the number of positive core numbers [22].

Despite these observations, the absence of direct comparison of Asian and Caucasian cohorts has meant that it has not been possible to understand whether race was an independent predictor of poor outcome, or whether there ware critical differences in the clinical and pathological features studies of Asian and Caucasian men that accounted for the apparent differences in rates of upgrading and upstaging. In multivariate logistic regression analysis in each race, age was not associated with upgrading or upstaging in Caucasians. Only prostate volume was associated with upgrading in Caucasians. However, age and prostate volume were associated with upgrading or upstaging in Koreans. The means of age and prostate volume were significantly different between two groups. Therefore, we analyzed the whole factors (age, PSA, prostate volume, race) in multivariate logistic regression.

One important difference between Asian and Caucasian radical prostatectomy cohorts that has not been carefully controlled for is that prostate volumes in Asians are smaller [22, 23]. Several studies have suggested that small prostate volume is associated with more aggressive behavior of prostate cancer, although the underlying reasons for this finding are poorly understood [24]. In a Korean cohort study, Chung et al found that smaller prostate volume was a predictor of Gleason score upgrading after radical prostatectomy [25]. From a Japanese study, Yashi et al reported that large prostate volume was a significant predictor for insignificant cancer on prostatectomy specimens with favorable pathologic features on biopsy [26]. Similar results have been reported in Western countries with primarily Caucasian populations. From the SEARCH database, Turley et al reported that larger transrectal ultrasound volumes were at decreased risk for clinically significant upgrading after radical prostatectomy [27]. In a large cohort of 4,500 Swedish patients, smaller prostate volume was associated with adverse pathology on multivariate analysis [11].

In our study, prostate volume was an important and independent predictor of upgrading and upstaging, implicating it, and not Asian race, as an important factor in the different rates of adverse pathology observed previously. It is noteworthy that the median PSA levels were similar in the Korean and Caucasian cohorts, and the PSA density (PSAD) was significantly higher in the Korean men. PSAD has been known to be associated with significant risk of upgrading in men presenting with low risk disease and has been used in some AS cohorts for patient selection.[4, 28] In a large multi-institutional AS cohort, PSAD was a strong and independent predictor of adverse reclassification in men on AS [5]. Given the strong association of PSAD with adverse outcome in patients who are candidates for AS, and the findings in our study, it appears to be critical for PSAD to be used in selection of Korean men with apparent low risk disease for enrollment in AS protocols.

Our study also implicates difference in age at diagnosis, PSA levels, and number of positive cores as additional factors that account for different rates of adverse pathology in Asian men. In Korea, like many Asian countries, systematic PSA screening for prostate cancer has not been practiced as in the United States. As a result, men in Korea were significantly older at the time of diagnosis in our series. Older age has been independently associated with an increased risk of upgrading at radical prostatectomy in men who are candidates for active surveillance [29]. However, in this series older age was not associated with worse RFS or OS, suggesting that it might be less critical to consider age in offering men AS for low risk prostate cancer. Additional work will be necessary to assess the effects of age and outcome in low risk Asian men.

The present study has some limitations that need to be mentioned. Our study is retrospective and with a relatively small sized cohort who all underwent treatment, which limits our ability to predict outcomes if these men had elected for active surveillance. In addition, there was no central pathology review despite inter- or intraobserver variability in Gleason grading, although both sites had experienced genitourinary pathologists who performed grading in accord with ISUP 2005 criteria [30]. We did not have detailed information on the extent of tumor involvement per core (percentage or total length) at biopsy and tumor volume after radical prostatectomy. However, both sites have detailed and complete clinical and pathological databases that allowed direct comparison of the two populations with a high level of granularity.

## Conclusions

Through a direct comparison of prostatectomy pathology from Korean and Caucasian men who qualify for active surveillance, we found that neither upgrading nor upstaging were associated with race. Importantly, prostate volume, PSA levels, number of positive cores and age were significant predictors for upgrading or upstaging in men regardless of race. Since Asian men presented with similar median PSA levels and smaller prostates than Caucasian men, our findings strongly suggest that PSAD is an important feature to assess in Asian men considering active surveillance, and to a large extent accounts for the higher rates of upgrading and upstaging observed previously in radical prostatectomy series from Asia.
